# *De Novo* Transcriptional Analysis of Alfalfa in Response to Saline-Alkaline Stress

**DOI:** 10.3389/fpls.2016.00931

**Published:** 2016-07-04

**Authors:** Yi-Min An, Li-Li Song, Ying-Rui Liu, Yong-Jun Shu, Chang-Hong Guo

**Affiliations:** State Key Laboratory of Molecular Genetics, Harbin Normal UniversityHarbin, China

**Keywords:** *Medicago sativa*, ion torrent sequencing, transcription factors, peroxidase, flavonol, saline-alkaline stress

## Abstract

Saline-alkaline stress, caused by high levels of harmful carbonate salts and high soil pH, is a major abiotic stress that affects crop productivity. Alfalfa is a widely cultivated perennial forage legume with some tolerance to biotic and abiotic stresses, especially to saline-alkaline stress. To elucidate the mechanism underlying plant saline-alkaline tolerance, we conducted transcriptome analysis of whole alfalfa seedlings treated with saline-alkaline solutions for 0 day (control), 1 day (short-term treatment), and 7 days (long-term treatment) using ion torrent sequencing technology. A transcriptome database dataset of 53,853 unigenes was generated, and 2,286 and 2,233 genes were differentially expressed in the short-term and long-term treatment, respectively. Gene ontology analysis revealed 14 highly enriched pathways and demonstrated the differential response of metabolic pathways between the short-term and long-term treatment. The expression levels of 109 and 96 transcription factors were significantly altered significantly after 1 day and 7 days of treatment, respectively. Specific responses of peroxidase, flavonoids, and the light pathway component indicated that the antioxidant capacity was one of the central mechanisms of saline-alkaline stress tolerance response in alfalfa. Among the 18 differentially expressed genes examined by real time PCR, the expression levels of eight genes, including inositol transporter, DNA binding protein, raffinose synthase, ferritin, aldo/keto reductase, glutathione *S*-transferase, xyloglucan endotrans glucosylase, and a NAC transcription factor, exhibited different patterns in response to saline and alkaline stress. The expression levels of the NAC transcription factor and glutathione *S*-transferase were altered significantly under saline stress and saline-alkaline stress; they were upregulated under saline-alkaline stress and downregulated under salt stress. Physiology assays showed an increased concentration of reactive oxygen species and malondialdehyde and a decreased content of chlorophyll, indicating that anti-oxidation and detoxification play an important role in response to saline-alkaline stress. Overall, the transcriptome analysis provided novel insights into the saline-alkaline stress tolerance response mechanisms in alfalfa.

## Introduction

Abiotic stresses, including drought, salinity, and extreme temperatures, adversely affect crop production worldwide. Soil salinity and alkalinity are among the major abiotic factors that limit crop yield. More than 954 million hectares of the global land surface consist of sodic soil (Zhang et al., 2006). In the Song-nen Plain, the main crop production area of Northeast China, approximately 3.78 million hectares of land are threatened by soil salinization and alkalization (Yang and Wang, 2015). Sodic soils with high pH and salt concentration restrict plant growth and limit agricultural production in this area.

Plant damage caused by saline stress is usually divided into three main categories: osmotic shock, toxic cation stress, and high pH damage. High salt concentrations reduce the soil water potential, resulting in osmotic shock (Wong et al., 2006), which is a state when an excess of sodium ions inhibits the metabolic processes in the cells and generates secondary metabolites such as peroxides (Zhu, 2001). Numerous studies have focused on plant salt tolerance; however, information on the combined saline and alkaline stress (hereafter referred to as saline-alkaline stress) is still limited. Thus, the gene expression regulation and molecular mechanisms involved in plant saline-alkaline stress tolerance are yet to be discovered. Saline-alkaline stress shares many factors with saline stress, such as excess of Na^+^ and water deficit, that trigger similar responses in both stress types (Gong et al., 2014). However, alkaline stress is unique for its high pH levels that initiate the synthesis of reactive oxygen species (ROS) and malondialdehyde that damage the plant cell membrane and intracellular components. High pH due to soil alkalization is a crucial and critical stress condition compared with salinity stress, because increased ion concentrations have more adverse effects on plant growth than soil salinization alone. Therefore, alkaline stress is a more diversified form of stress, and plant tolerance to alkalinity reflects a more complex tolerance mechanism (Peng et al., 2008).

Plant saline-alkaline stress tolerance mechanisms involve the expression of a cluster of genes and the interaction among gene products rather than the expression of individual genes (Xu and Tuyen do, 2012; Gong et al., 2013). Gene expression is affected by many internal and external factors (Yang et al., 2009); hence, a more comprehensive understanding of saline-alkaline stress tolerance mechanisms in plants should be inferred at the gene expression level. Transcriptome analysis is a novel tool for the study of plant saline-alkaline stress tolerance mechanisms (Sun et al., 2013; Wang C. et al., 2014) at the RNA level and uncovers the differences in common stress response pathways as a response to a variety of stresses such as saline, peroxide, and saline-alkaline stress.

In recent years, high throughput technologies have been rapidly developed and used in the genome-wide gene expression profiling of *Arabidopsis* (Jiang and Deyholos, 2006), rice (Kawasaki et al., 2001), wheat (Kawaura et al., 2006), and soybean (Irsigler et al., 2007; Ge et al., 2010). Next generation sequencing is a large-scale analysis method of differential gene expression using various platforms (Genome Analyzer [Solexa/Illumina], 454 [Roche], ABI-SOLiD [Applied Biosystems], and HeliScope [SeqLL LLC, Woburn, MA, USA]) that facilitate RNA-seq analysis and unravel a variety of stress responses on a transcriptome-wide scale in non-model plant species. Ion torrent sequencing detects hydrogen ions that are released during DNA polymerization, and it differs from other sequencing technologies, since it does not employ any modified nucleotides or optics. This technology is a rapid, compact, and economical sequencing system that can be used for plant transcriptome analysis.

Alfalfa (*Medicago sativa*) is a major forage legume cultivated worldwide, including the Song-nen Plain, Northeast China (Li et al., 1996). ‘Zhaodong’ is an alfalfa cultivar with resistance to various abiotic stresses such as freezing, drought, salinity, and saline-alkaline stress (Peng et al., 2008). Despite its economic importance, the physiological and molecular mechanisms underlying its response to abiotic stresses are poorly understood. The completed sequencing project of *M. truncatula*, a close relative of *M. sativa*, provides a reference genome to identify tentative consensus sequences in alfalfa in response to abiotic stress and generate an expression pattern of stress response.

In this paper, we conducted transcriptional profiling of the whole seedling alfalfa treated with saline-alkaline solution using ion torrent to assess changes in gene expressions. Differentially expressed gene (DEG) profiles as responses to stress were presented, and DEG response to saline-alkaline stress was annotated. DEG-specific responses to saline-alkaline conditions were identified through comparison with the saline stress response pathway, and specific mechanisms of saline-alkaline stress tolerance were elucidated. The transcriptome analysis of alfalfa in response to saline-alkaline stress might provide novel insights into plant saline-alkaline stress tolerance mechanism based on DEGs and reveal the related pathway of plant saline-alkaline tolerance.

## Materials and Methods

### Plant Materials and Treatment Conditions

Seeds of Zhaodong, supplied by the Heilongjiang Animal Science Institute, China, were germinated in pots with distilled water for 2 days in the dark, transferred to vermiculite with ½ Hoagland solution, and kept in a culture room for 4 weeks at 60% relative humidity, 22°C, and a photoperiod of 16 h light/8 h dark.

Four-week-old seedlings were treated with ½ Hoagland solution, containing 0.1 M mixed alkaline solution (Na_2_CO_3_: NaHCO_3_, 1: 2) for 0 day (control), 1 day (short-term treatment), and 7 days (long-term treatment). The harvested whole seedlings from each treatment were frozen in liquid nitrogen and stored at –80°C for RNA-seq, qRT-PCR, and physiological analyses. Ion torrent sequencing was conducted by BGI (Shenzhen, China) in two replicates for each sample.

The same batch of whole-seedling samples was treated with 0.1 M mixed alkaline and 0.1 M NaCl for 7 days prior to qRT-PCR in order to identify the differences in response mechanisms under saline stress and saline-alkaline stress conditions.

### RNA Extraction and Transcriptome Sequencing

Transcriptome sequencing was completed by BGI using Ion Proton I RNA-seq (Thermo-Fisher Scientific, Waltham, MA, USA). Total RNA was extracted from the control and samples treated with 0.1 M mixed saline-alkaline solution using One Step RNA Reagent (Bio Basic Inc., Markham, ON, Canada), according to the manufacturer’s protocol, and purified using the RNeasy Plant Mini Kit (Qiagen, Valencia, CA, USA). The whole transcriptome cDNA library was prepared using the Ion Total RNA-Seq kit V2 (Life Technologies Corp., Carlsbad, CA, USA). Double-stranded cDNA was ligated to barcoded adapters and then sequenced using an Ion PI^TM^ Chip (Ion torrent, Life Technologies, Carlsbad, CA, USA) by BGI. Raw data processing, adapter sequence removal, base-calling, and quality value calculations were performed using Torrent Suite^TM^ 4.0 (Life Technologies, Carlsbad, CA, USA). Quality reads were obtained by trimming the raw reads at a minimum PHRED score *Q* = 20 (Shu et al., 2015).

### Reads Assembly and Functional Annotation

Total reads obtained by Ion torrent sequencing were assembled by Trinity assembler (Grabherr et al., 2011), and redundant reads were removed using iAssembler (Zheng et al., 2011). The contigs that presented zero transcript expression (RPKM) were adjusted to 1 in order to avoid negating the subsequent calculation of fold change. The differentially expressed transcripts were identified based on fold changes (FC ≥ 2 or FC ≤ 0.5) and statistical analysis (*p* ≤ 0.001) using edgeR package in R (R team, Vienna, Austria).

DEGs were annotated using two methods: (1) Reads generated by transcriptome analysis were mapped onto *M. truncatula*, a species with a completely sequenced genome (Mt4.0 data release; Tang et al., 2014) highly homologous to alfalfa, using Bowtie 0.12.7 (Langmead et al., 2009); (2) Functional annotation of unigenes assembled by Trinity was conducted using BLASTx search against the Nr and Swiss-Prot databases and *M. truncatula*, *Arabidopsis thaliana*, and *Glycine max* as reference sequences at a cut-off E-value of 10e-5. The unigenes were assigned to known functional groups and biological processes using Gene Ontology (GO) tools (Ye et al., 2006)^1^.

Transcription factors were searched and identified from unigenes using iTAK^2^. Venny tool was used for drawing and comparison of Venn diagrams (Oliveros, 2007–2015).

### Quantitative Reverse Transcription PCR (qRT-PCR) Analysis

Total RNA was extracted from the same group of samples that were used in transcriptome analysis using an RNA extraction kit (TianGen Biotech, Beijing, China). RNA was reverse-transcribed into cDNA using the PrimeScript™ RT reagent kit (TaKaRa, Shiga, Japan). Twenty primers (20 random genes and 20 stress-induced genes) were used in real-time RT-PCR, and data were normalized using the actin gene.

The reaction mixture (20 μl) contained 10 μl of SYBR Green RealTime PCR Master Mix (Toyobo, Osaka, Japan), 2 μl of cDNA template, and 0.5 μM of each forward and reverse primer. The amplification was run in an ABI 7300 sequencer with the following cycling parameters: initial denaturation at 94°C for 30 s, followed by 45 cycles at 94°C for 15 s, 55°C for 30 s, and 72°C for 30 s. Real-time PCR was carried out in triplicate to confirm the accuracy of the results. All the relative expression levels were log_2_-transformed.

### Measurement of Physiological Parameters under Saline-Alkaline Stress

The levels of H_2_O_2_ and ${\text{O}}_{2}^{-}$ were measured as described by Mukherjee and Choudhuri (1983) and Liu and Pang (2010), respectively. To measure the ${\text{O}}_{2}^{-}$ content, plant material was extracted in potassium phosphate buffer (pH 7.8) and then, centrifuged at 10,000 rpm for 5 min. After incubating the supernatant at 25°C for 20 min, 17 mM sulfanilamide and 7 mM *a*-naphthylamine were added, and the mixture was incubated at 25°C for additional 20 min. The absorbance was read at 530 nm, and the ${\text{O}}_{2}^{-}$ content was calculated against the standard curve prepared with NaNO_2_. To determine the H_2_O_2_ content, plant tissue was extracted with cold acetone (4°C) and mixed with 0.1% TiCl_4_ and ammonia. The mixture was centrifuged at 10,000 rpm for 10 min. The precipitate was dissolved in 2 mM H_2_SO_4_ and the absorbance was read at 415 nm; the H_2_O_2_ content was calculated using the standard curve.

Peroxidase (POD) activity was measured according to the protocol described by Gong (2001). The concentration of malondialdehyde (MDA) was measured as described by Hodges et al. (1999). Plant tissue was extracted in precooled acetocaustin and centrifuged at 10,000 rpm for 10 min. The supernatant was mixed with thiobarbituric acid and transferred to a boiling water bath for 15 min. After centrifuging at 10,000 rpm for 10 min, the concentration of MDA was calculated from the absorbance read at 532 nm and 450 nm.

The chlorophyll content was measured as previously described (Gong et al., 2013). Aldo/keto reductase (AKR) activity was determined as described by Wang Q. et al. (2014). Root tissues exposed to saline stress or saline-alkaline stress for 7 days were extracted in cold potassium phosphate buffer (pH 7.4) and centrifuged at 12,000 rpm for 10 min. The supernatant was added to a mixture of 5 mM D-glucuronic acid and dimethylsulfoxide, which was previously incubated at 30°C for 5 min, and 10 mg mol^−1^ NADPH. The absorbance was read at 532 nm every minute for 3 min. The activity of AKR was calculated as follows: u/g = ΔA × V/(6.22 × M).

## Results

### Ion Torrent Sequencing and Reads Assembly

After removing low-quality adaptor and barcode sequences, 16,209,292; 14,444,593; and 19,940,365 draw reads were obtained from the control, the short-term treatment, and the long-term treatment, respectively. Of these, 15,774,180; 14,109,229; and 19,355,510, respectively, were clean reads (**Table 1**). The percentage of clean reads was 97.67% and 97.24% in the short-term treatment and the long-term treatment, respectively, indicating a high sequencing quality. Contigs from all samples were assembled into 53,853 unigenes that were used to generate a non-redundant unigene library for further analysis of the *M. sativa* transcriptome under saline-alkaline stress.

**Table 1:** *De novo* transcriptome assembly of contig sequences of alfalfa exposed to saline-alkaline stress for different length of time.

	1 Day	7 Days
Total reads	14,444,593	19,940,365
Clean reads	14,109,229	19,355,510
Clean reads percentage	97.67%	97.24%
Unigenes	53,924	53,854

### Identification of DEG and Functional Classification

**FIGURE 1:**
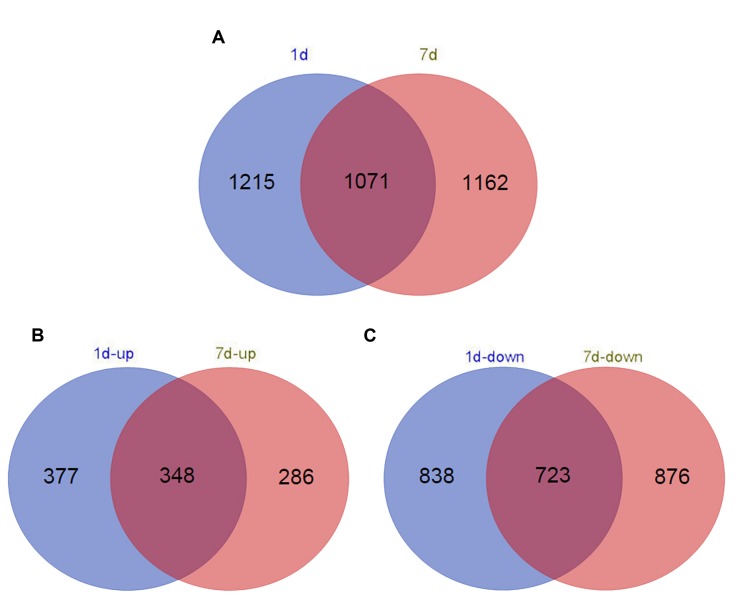
**Venn diagram of the number of differentially expressed genes (DEGs) (log_2_ > 1.5) in different time points in *de novo* transcriptome (false discovery rate < 0.025). (A)** Distribution of all DEG **(B)** up-regulated DEG; and **(C)** down-regulated DEG annotated in samples treated with alkaline-solution for 1 day (red circles) and 7 days (blue circles).

Functional annotation of non-redundant unigenes against the Nr database and the Swiss-Prot database using BLASTx resulted in 2,286 (1 day treatment) and 2,233 (7 days treatment) DEGs (log_2_ > 1.5, *p* < 1e-5; Supplementary Table S1). In total, 1,561 upregulated DEGs and 725 downregulated DEGs were identified in the short-term treatment, whereas 1,599 upregulated DEGs and 634 downregulated DEGs were identified in the long-term treatment (**Figure 1**).

### qRT-PCR Conformation of Ion Torrent Sequencing

To validate transcriptome results, 40 genes (20 random and 20 stress response genes) differentially expressed in the short-term treatment and the long-term treatment were selected. The qRT-PCR results were consistent with the transcriptome sequencing results (**Table 2**).

**Table 2:** Genes used for validation of next generation sequencing (NGS) data by quantitative reverse transcriptase PCR (qRT-PCR).

Gene	qRT-PCR		NGS	
	1 Day	7 Days	1 Day	7 Days
Medtr4g020110.1	6.35	1.13	5.65	8.07
Medtr1g066380.1	5.63	3.29	4.25	3.86
Medtr7g085220.1	5.56	1.75	2.92	1.25
Medtr8g061360.1	5.53	1.58	4.37	1.39
Medtr7g069980.1	5.15	2.03	3.76	2.59
Medtr7g079080.1	4.96	1.18	5.95	4.03
Medtr1g090060.1	4.69	2.34	5.93	4.31
Medtr3g467100.1	4.58	3.61	4.58	4.31
Medtr5g055690.1	4.50	5.01	1.73	1.40
Medtr7g112560.1	4.40	2.72	3.10	3.36
Medtr2g049020.1	4.27	2.32	3.47	4.62
Medtr6g045403.1	3.69	5.08	4.07	4.76
Medtr4g021350.1	3.19	1.27	2.50	2.47
Medtr7g080900.1	2.14	2.41	5.26	3.81
Medtr5g079730.1	0.45	0.60	0.54	3.94
Medtr4g101820.1	−0.65	1.95	−0.69	−0.02
Medtr4g099340.1	−1.77	−0.52	−2.03	−1.78
Medtr4g076600.1	−2.44	−3.52	−1.66	−1.78
Medtr7g115270.1	−3.90	−6.87	−3.32	−2.58
Medtr7g007120.1	−4.39	−1.05	−2.77	−2.47

The most highly upregulated genes were delta-1-pyrroline-5-carboxylate synthetase (Medtr4g020110.1), encoding one of the key enzymes of proline biosynthesis, glutathione *S*-transferase (Medtr1g090060.1) that participates in the non-enzymatic elimination pathway of ROS, and flavonol synthase (Medtr5g055690.1) that is a key enzyme of flavonoid biosynthesis. The most highly downregulated genes were the light-harvesting protein (Medtr4g099340.1) and the light-harvesting complex I chlorophyll A/B-binding protein (Medtr6g011880.1) that play an important role in the capture of photons in the light pathway, the auxin-binding protein ABP19a (Medtr8g020590.1), and F-box (Medtr7g115270.1).

**FIGURE 2:**
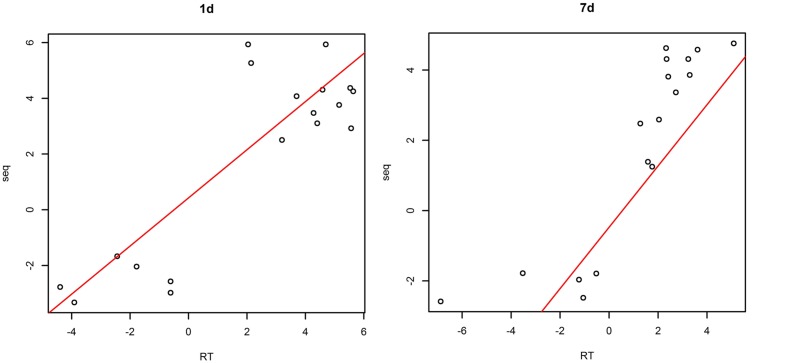
**Validation of next generation sequencing (NGS data by quantitative reverse transcriptase PCR (qRT-PCR) in samples treated with alkaline-saline solution for 1 day and 7 days**.

The plotting of RT-PCR and Ion torrent transcriptome data (**Figure 2**) showed a high correlation coefficient (*R*^2^ = 0.732, *P* < 0.05 in samples treated with saline-alkaline solution for 1 day and *R*^2^ = 0.756, *P* < 0.05 in samples treated with saline-alkaline solution for 7 days) between the two data sets, whole data see Supplementary Table S2.

### GO Analysis

**FIGURE 3:**
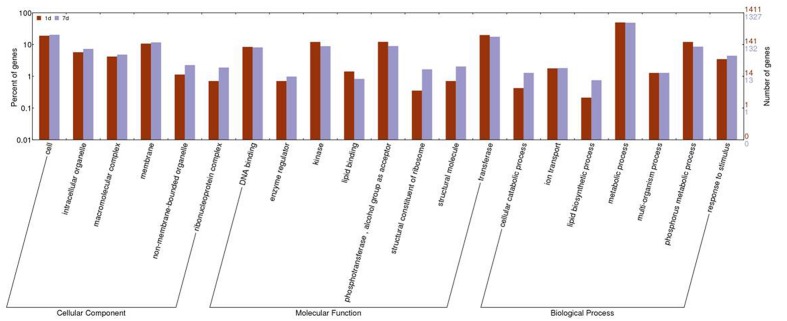
**Histogram of gene ontology (GO) categories in response to 1 day and 7 days of treatment with saline-alkaline stress**. The bars represent log_2_-transformed ratio between category portions in up-regulated and down-regulated gene sets.

The GO cluster analysis of DEGs obtained from the two treatments was assembled using WEGO. The expression of genes involved in binding, antioxidant activity, membrane, and transport was similar between the two treatments. Subcategories of the biological process category, such as biopolymer modification, phosphate metabolism, lipid biosynthesis, and cellular macromolecule catabolism, were changed significantly between the two treatments. In the category of molecular functions, transferase activity, kinase activity, phosphotransferase activity, and structural constituent of ribosome had significantly different expression levels. In the category of cell components, ribosome, intracellular non-membrane-bound organelle, and cytosol were significantly different between the two treatments (**Figure 3**; Supplementary Table S3).

### Gene Differential Expression in Short-Term and Long-Term Treatments

**FIGURE 4:**
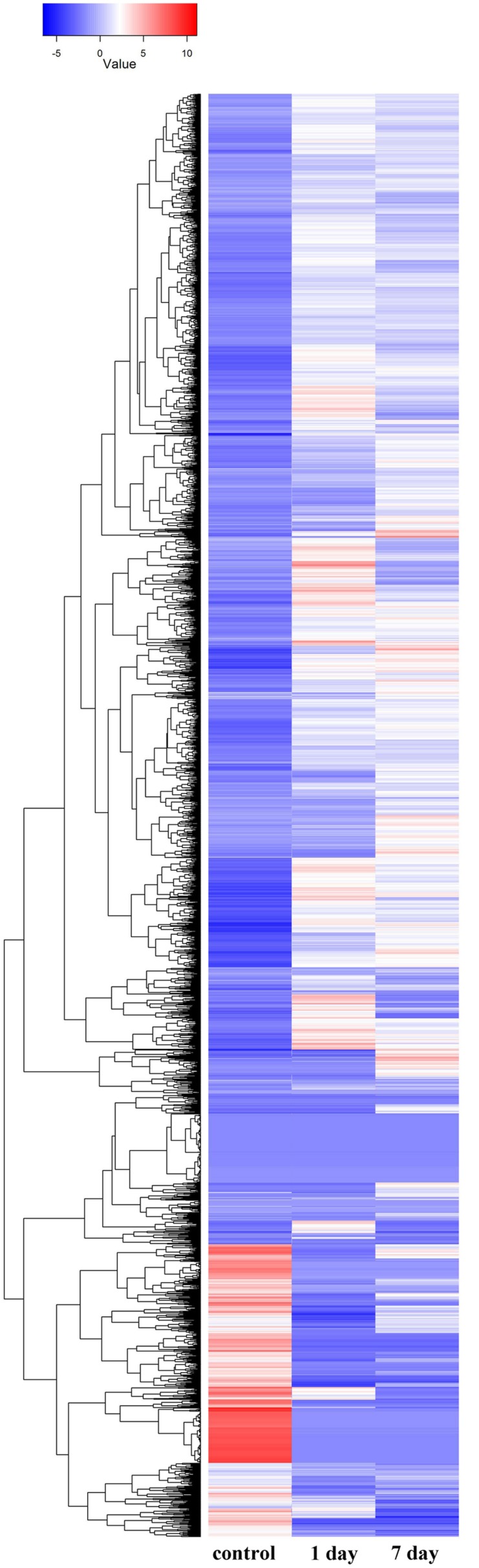
**Hierarchical cluster analysis of differentially expressed genes based on expression pattern of 53,853 genes derived from the control, 1 day and 7 days treatments**.

Gene ontology cluster analysis of the 53,853 unigenes showed that under alkaline-saline stress conditions, the expression levels of DEGs as well as the differential expression of various gene types were modified (**Figure 4**; Supplementary Table S4).

In the molecular function category, the expression levels of genes clustered in GO categories, such as protein kinase, structural molecule activity, transferase, and POD, were altered significantly. Among these, protein kinase, phosphotransferase, and transferase had higher expression levels in the short-term treatment than in the long-term treatment. These results indicated that the metabolic processes were mediated by kinase, and that the efficiency of intracellular transporter was greater at on day 1 of treatment than at day 7 of treatment. The activity of transcription factor was more enhanced at day 7 than at day 1 of treatment, whereas the expression level of POD was high in both treatments.

Genes involved in the structural constituent of the ribosome subcategory did not alter their expression in the short-term treatment; however, they were highly induced in the long-term treatment, showing that more ribosomes were assembled to translate proteins. These results suggested that intracellular homeostasis does not form in plant cells that are adapted to saline-alkaline stress, unless new homeostasis is established to meet the high expression levels of the induced genes.

Lipid biosynthetic process, a subcategory of the biology process category, had significantly more expressed genes in the long-term treatment than in the short-term treatment. The DEGs in the lipid biosynthetic process subcategory were focused on the expression and modification of phospholipids at day 1 of treatment and the transport and metabolism of lipids at day 7 of treatment with the sustained expression of inositol.

### Differential Expression of Peroxidase under Saline-Alkaline Stress

In the transcriptome category, BLASTx against *M. truncatula* genome using *de novo* assembly clustered PODs into three classes: POD family protein, cationic POD, and class III POD. In addition, a few genes clustered into respiratory burst oxidase-like protein D, POD family protein, and cationic POD, which belong to class I POD and were induced by the short-term treatment and upregulated in the long-term treatment. For instance, c67928_g1_i1 (similar to Medtr2g008730.1) and c70153_g1_i3 (similar to Medtr1g066380.1) were both strongly induced and upregulated by stress, reaching the expression levels of 4.766 and 4.252, respectively. In the present study, the expression of the vast majority of class I POD was lower in the long-term treatment than in the short-term treatment, yet still higher than in the control. In contrast, the member of class III POD c69915_g1_i1 (similar to Medtr7g072510.1) was upregulated under stress, increasing the levels 4.53-fold at day 7 of treatment compared with the levels observed at day 1 of treatment.

Although the majority of PODs were upregulated under stress conditions, a different expression tendency was observed in few PODs. For instance, c41991_g1_i1 (similar to Medtr4g046713.1) and c60608_g1_i1 (similar to Medtr6g043460.1) were downregulated at day 1 of treatment. However, the metabolic pathway and specific functions of these genes remain unknown because of the limited annotation information.

### Flavonoids Metabolism-Related Genes Were Induced

The pathway of flavonoid biosynthesis and metabolism is well understood (Winkel-Shirley, 2002). Flavonoids participate in ROS elimination as electron donors (Hernández et al., 2009). In addition, flavonoids bind metal ions and thus reduce the damage caused by metal ions (Pourcel et al., 2006). Flavonoids are also signal molecules that interact with transcription factors, kinases, and histones to regulate gene expression at different levels (Ithal and Reddy, 2004).

In transcriptome data, the expression of several rate-limiting enzymes and modified genes in the flavonoid metabolic pathway was significantly altered. The key enzymes of the flavonoid biosynthesis pathway, including the flavonol synthase c68976_g2_i1 (similar to Medtr5g055690.1) and the chalcone synthase c69319_g1_i1 (similar to Glyma.08g109400.1), as well as the pathway intermediate enzyme 4-*p*-coumaric acyl CoA ligase c80508_g1_i1 (similar to Medtr1g073180.1) were upregulated under stress. *p*-Coumaric acyl CoA is the substrate in the synthesis of chalcone, which limits the rate of flavonoid biosynthesis. Furthermore, the flavonoid glycosyltransferase c71058_g2_i1 (similar to Medtr8g083290.1) and the 2-hydroxy isoflavone dehydratase c69844_g1_i1 (similar to Medtr1g105020.1) were similarly upregulated. Glycosylation increases the water solubility of flavonoids and induces other modifications such as acylation (Schwinn et al., 1997). Acylation enhances the stability of flavonoids in the cytoplasm. A previous study confirmed the presence of acylated flavonoids in the chloroplast, where they prevent peroxidation of the thylakoid membrane on the oxidizing side of the Photosystem II (PSII). Isoflavone, a substrate of various flavonoids, is formed by dehydration of 2-hydroxy isoflavone in a reaction catalyzed by 2-hydroxy isoflavone dehydratase. The upregulation of 2-hydroxy isoflavone dehydratase may suggest a high synthesis rate of isoflavone (Hernández et al., 2009).

### Downregulation of the Light Pathway

Plants reduce the photosynthesis rate and delay their growth under both salinity stress (Zhang and Shi, 2013) and alkaline stress (Li et al., 2010). A previous study showed that the synthesis of the photosynthetic pigments is blocked by abiotic stressors such as salinity (Zhang and Shi, 2013) and freezing temperatures. In the present study, the light-harvesting complex I chlorophyll *a*/*b*-binding protein c73857_g2_i1 (similar to Medtr6g011880.1) was significantly downregulated in response to saline-alkaline stress, suggesting an outstanding inhibition of the plant light-harvesting system. Moreover, the light-harvesting proteins c66633_g1_i1 (similar to Medtr3g101670.1) and c73420_g1_i1 (similar to Medtr4g099340.1) were downregulated, suggesting the inhibition of the photosynthetic system. Nevertheless, a previous study on the transcriptome analysis of *Eleusine coracana* subjected to salinity stress reported the low efficiency of PSII, similar to the behavior of the chlorophyll *a*/*b* binding protein and the light-harvesting proteins under alkaline-saline stress conditions (Rahman et al., 2014).

The expression level of ribulose bisphosphate (RuBP) carboxylase (similar to Medtr7g007120.1), a key enzyme of carbon assimilation in the light pathway, declined and remained low under stress. RuBP carboxylase catalyzes the carboxylation of RuBP, initiating the process of carbon assimilation. The downregulation of RuBP carboxylase indicates that the light pathway is inhibited under saline-alkaline stress and may reduce the generation of ROS. Charge separation of the photon-excited PSII reaction center Chl (P680) is suppressed, leading to the accumulation of excited triplet P680, which reacts with O_2_ producing singlet O_2_ (^1^O_2_) (Gong et al., 2013).

### Differential Expression of Lipid Metabolism

The lipid metabolism subcategory mainly clustered in GO categories related to synthesis and modification of the calcium signal pathway in the short-term treatment. However, this cluster also included the synthesis and transport of lipids in addition to the calcium signal pathway in the long-term treatment.

The calcium signal pathway plays an important role in the biotic and abiotic stress response (Xiong et al., 2002). Various calcium-dependent stress response proteins are regulated by this pathway as well as the transcription factors are regulated by calcium and calcium binding promoters. The *myo*-Inositol 1-phosphate synthase c52088_g1_i1 (similar to Medtr3g087590.2), which is a component of the calcium signal pathway, was upregulated, increasing the expression levels four-fold in the long-term treatment compared with those in the control and short-term treatment. Similarly, other components of the calcium pathway, such as Medtr2g095220.1, Medtr7g038700.2, and Glyma.08G109400.1.p maintained high expression levels in the long-term treatment, suggesting that the calcium signal pathway remains active during continuous stress. In a similar study on salt stress, phosphate inositol was upregulated significantly when treated with NaCl for 7 days (Postnikova et al., 2013).

### Differentially Expressed Transcription Factors in Transcriptome

**FIGURE 5:**
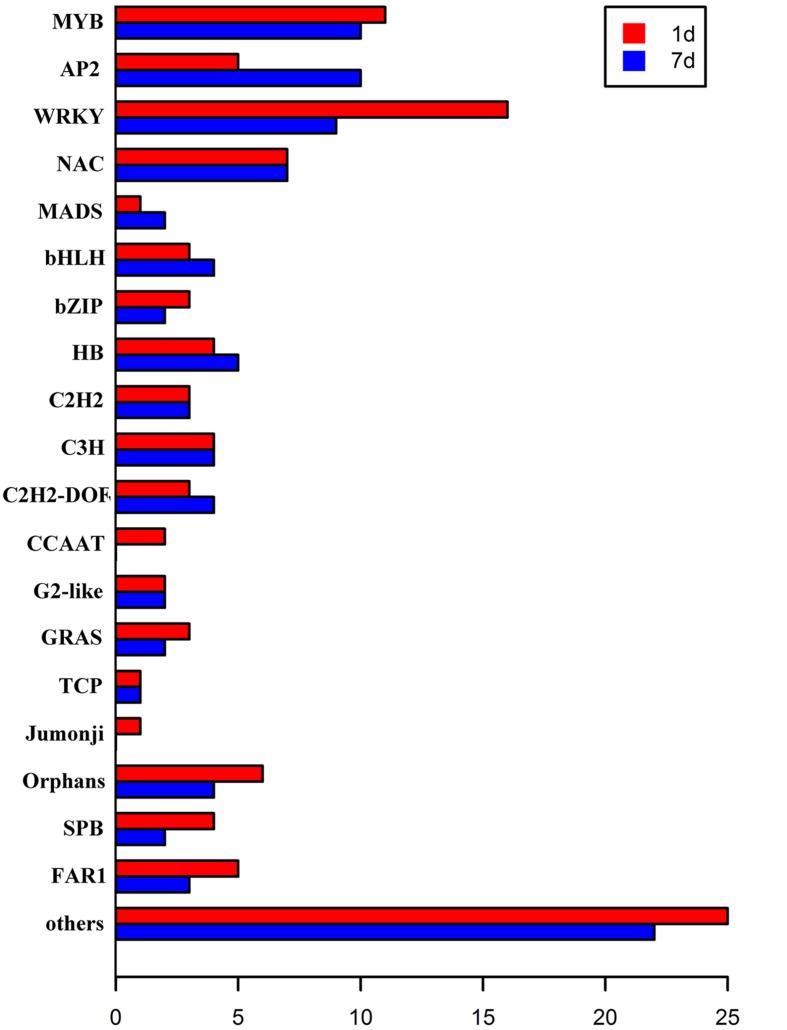
**Transcription factors differentially expressed in samples after 1 day and 7 days of saline-alkaline stress**.

Transcription factors regulate the expression of genes involved in plant cell metabolism and play an important role in response to environmental stress, including stress response pathways, growth processes, morphogenesis, and responses to various stimuli. *De novo* assembly data contained 2,375 transcription factors homologous to *M. truncatula*. Moreover, the expression levels of 150 transcription factors (6.31% of all transcription factors) were significantly modified (log_2_ > 1.5) under stress (**Figure 5**). Of these, 109 transcription factors responded to saline-alkaline stress at day 1 of treatment and 96 at day 7 of treatment, including the major transcription factor families that respond to abiotic stress such as MYB, WRKY, NAC, AP2/EREBP, bHLH, and bZIP (**Table 3**; Supplementary Table S5).

**Table 3:** Transcription factors differentially expressed in samples after 1 day and 7 days of saline-alkaline stress.

TF family	1 Day	7 Days
MYB	11	10
AP2	5	10
WRKY	16	9
NAC	7	7
MADS	1	2
bHLH	3	4
bZIP	3	2
HB	4	5
C2H2	3	3
C3H	4	4
C2H2-DOF	3	4
CCAAT	2	0
G2-like	2	2
GRAS	3	2
TCP	1	1
Jumonji	1	0
Orphans	6	4
SBP	4	2
FAR1	5	3
Other	25	22
Total	109	96

*TF, transcription factor.*

The MYB transcription factor family presented the most significant changes under continuous stress. Of 179 MYB transcription factors that responded to saline-alkaline stress, 10 MYB were altered significantly at day 1 of treatment and 11 at day 7 of treatment. Although the majority of MYB family transcription factors showed stable upregulation trends in the long-term treatment, a few genes, including c70259_g1_i1 (similar to Medtr3g110028.2) and c30131_g1_i1 (similar to Medtr5g010650.1), were downregulated. In previous studies, MYBs were strongly upregulated in response to salinity in *Arabidopsis thaliana* (Xu et al., 2015) and *Glycine max* (Liao et al., 2008). In the transcriptome analysis of alfalfa, MYB was the secondary transcription factor family with the strongest response to salinity stress after AP2 (Postnikova et al., 2013). The expression of MYBs in alfalfa induced by saline-alkaline stress appeared similar to that under salinity stress, suggesting the important role of MYB in alfalfa saline-alkaline tolerance.

Of the 87 differentially expressed WRKY family transcription factors, 16 exhibited significant differential expression in the short-term treatment: 15 were upregulated and only c73314_g1_i1 (similar to Medtr7g109600.1) was downregulated. Among the transcription factors in the long-term treatment, c62895_g1_i1 (similar to Medtr3g095080.1), c67877_g1_i1 (similar to Medtr8g092010.1), c70411_g1_i1 (similar to Medtr4g132430.1), and c81562_g1_i4 (similar to Medtr5g094430.1) were moderately downregulated, except for c81562_g1_i4 that was upregulated. Among them, both WRKY33-like and WRKY75-like transcription factors were highly induced under saline-alkaline stress. A previous study revealed that WRKY33 is highly induced by salt stress and closely related to ABA sensitivity (Jiang and Deyholos, 2009), increasing *Arabidopsis* NaCl tolerance. WRKY75 was reported to be involved in regulating a nutrient starvation response and root development (Devaiah et al., 2007) and, through its co-expression with a GSTU family member, to limit plant growth and development as well as the level of GSTU expression. Additionally, Rushton et al. (2010) reported that the WRKY family of transcription factors was induced by salinity stress in *Arabidopsis thaliana*, confirming the important role of WRKY gene family in biological processes and responses to abiotic stress.

The NAC family comprises plant-specific transcription factors is one of the largest transcription factor families in plants involved in responses to many biotic and abiotic stresses (Nakashima et al., 2007, 2012). The members of the NAC transcription factor family showed similar expression in both treatments, whereas the expression of 11 NAC members was significantly modified in both treatments. An AtNAC47-like gene was highly upregulated under saline-alkaline stress, but downregulated under salt, drought, and ABA treatments. The high expression levels of NAC members indicated the important role of NAC in plant saline-alkaline stress tolerance.

The bHLH family of transcription factors was strongly induced by saline-alkaline stress. c77005_g1_i1 (similar to Medtr5g030770.1) was continuously upregulated until day 7 of treatment. The bHLHs are involved in the construction of the light pathway, in flavonoid biosynthesis, and specific root morphology.

The primary family of transcription factors was highly upregulated in the short-term treatment and moderately downregulated in the long-term treatment. Some transcription factors showed successive upregulation under stress, suggesting that these DETFs play an important role in plant tolerance to saline-alkaline stress.

### Non-annotated Genes

A total of 19,465 genes (36.14% of all unigenes) with complete open reading frame (ORF) were not annotated in the GenBank, and their expression levels were altered under stress. For instance, the putative protein Medtr1g100627 was upregulated 7.49-fold in the long-term treatment compared with the control, whereas the putative protein Medtr2g075340 was downregulated 7.53-fold in the short-term treatment. Several putative transmembrane genes, such as Medtr8g069283, Medtr2g022540, Medtr2g075940, Medtr1g014110, and Medtr5g095840, were altered significantly under stress, suggesting that plant saline-alkaline stress tolerance includes many unknown mechanisms.

### Specifically Expressed Genes under Saline-Alkaline Stress Compared with Saline Stress

**FIGURE 6:**
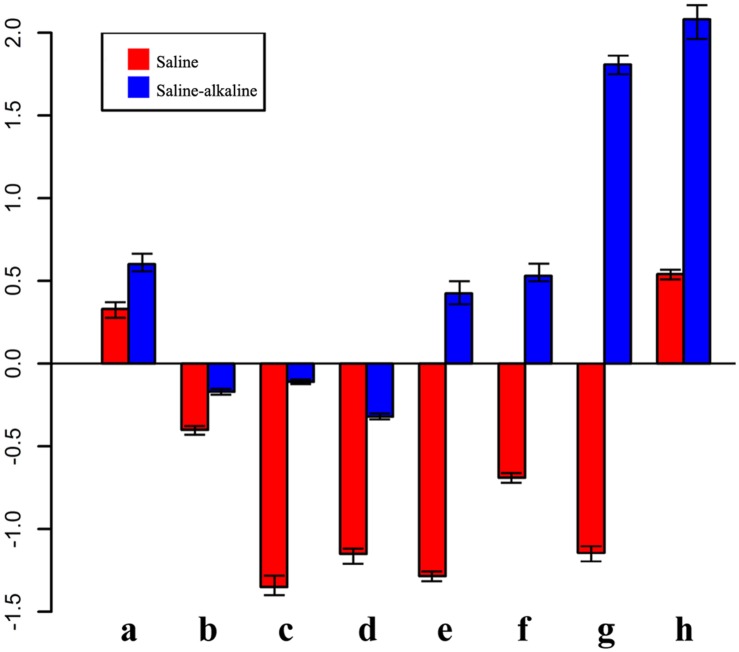
**Expression pattern of genes in response to saline-alkaline stress and saline stress after 7 days of treatment**. a, inositol transporter; b, DNA binding protein; c, raffinose synthase; d, ferritin; e, ACC synthetase; f, NAC transcription factor; g, glutathione *S*-transferase; h, xyloglucan endotransglucosylase.

The qRT-PCR results showed that the expression of eight genes significantly changed under saline and saline-alkaline stress for 7 days (**Figure 6**). These differences might suggest that plant tolerance to saline-alkaline environment involved a different mechanism from that in the tolerance to saline stress (Supplementary Table S6).

A group of genes showed higher expression in response to alkaline-saline stress than to salt stress. For instance, xyloglucan endotransglucosylase/hydrolase (similar to Medtr8g102220.1) was not enhanced under saline stress, but it was increased 2.08-fold by saline-alkaline stress, suggesting that the synthesis of the cell wall component was under stress.

Some genes were induced significantly by saline stress, but showed only a mild response to saline-alkaline stress under the same concentration of sodium. Raffinose synthase (Medtr4g115330.1) was downregulated 2.08-fold under saline stress, whereas the downregulation was –0.11 under saline-alkaline stress. The lower downregulation of raffinose synthase indicated that the raffinose synthase pathway is involved in the response to saline-alkaline stress in alfalfa. Similar regulation was also observed in the ferritin Medtr7g069980.1, suggesting that ferritin is always maintained at a certain level under saline-alkaline stress in the root because of its important role in the regulation of intracellular ion balance (Masuda et al., 2013).

Additional transcription factors responded differently under the two stresses. For instance, a member of the AP2 (Medtr4g100650.1) transcription factor family and an ERF transcription factor (Medtr7g085220.1) were downregulated under saline stress, but upregulated under saline-alkaline stress.

### Physiology Assay under Saline-Alkaline Stress

**FIGURE 7:**
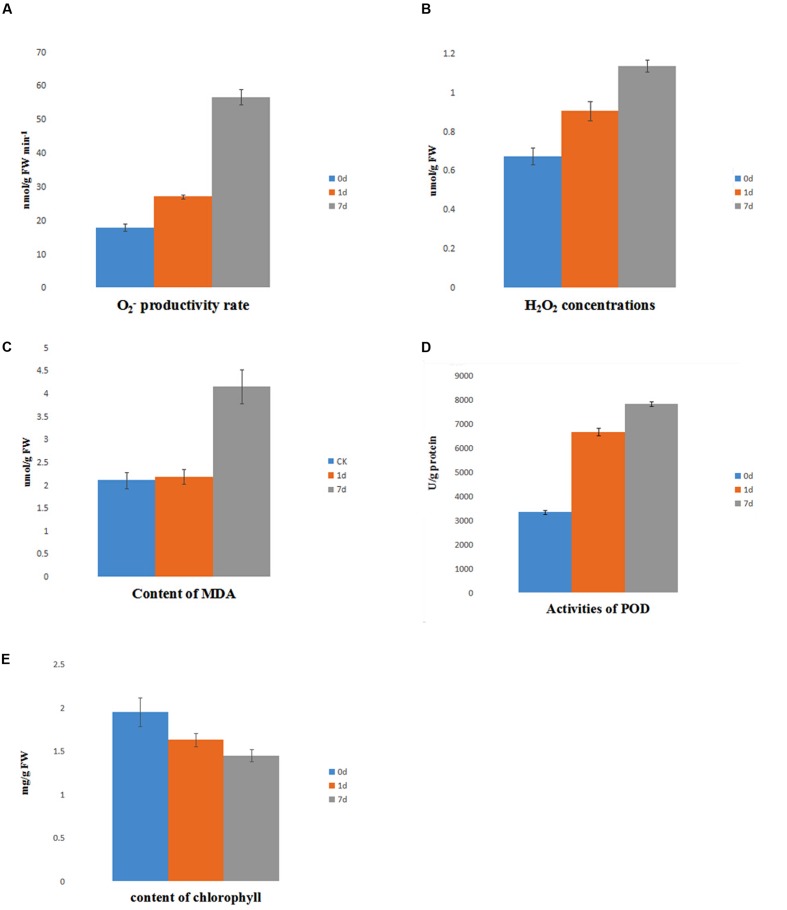
**Physiology assay of alfalfa under saline-alkaline stress. (A)** content of ${\text{O}}_{2}^{-}$; **(B)** content of H_2_O_2_; **(C)** content of MDA; **(D)** activity of POD; **(E)** content of chlorophyll.

The level of ROS is indicative of the physiological conditions in plants under saline-alkaline stress. The accumulation of ${\text{O}}_{2}^{-}$ showed a mild increase at day 1 of treatment, but it was markedly increased at day 7 of treatment. In contrast, the level of H_2_O_2_ gradually increased from day 0 to day 7 of treatment (**Figures 7A,B**). Furthermore, the activity of POD, which is strongly induced by ROS, was highly induced by saline-alkaline stress at day 1 of treatment and had a slightly higher activity at day 7 of treatment (**Figure 7D**). The content of MDA reveals the level of lipid peroxidation and indicates the damage to the membrane under stress. The accumulation of MDA had no marked change between day 0 and day 1 of treatment, but it increased at day 7 of treatment (**Figure 7C**).

The level of chlorophyll decreased under saline-alkaline stress. The total chlorophyll continued degrading (**Figure 7E**), suggesting a dramatic inhibition of chlorophyll synthesis due to saline-alkaline stress.

**FIGURE 8:**
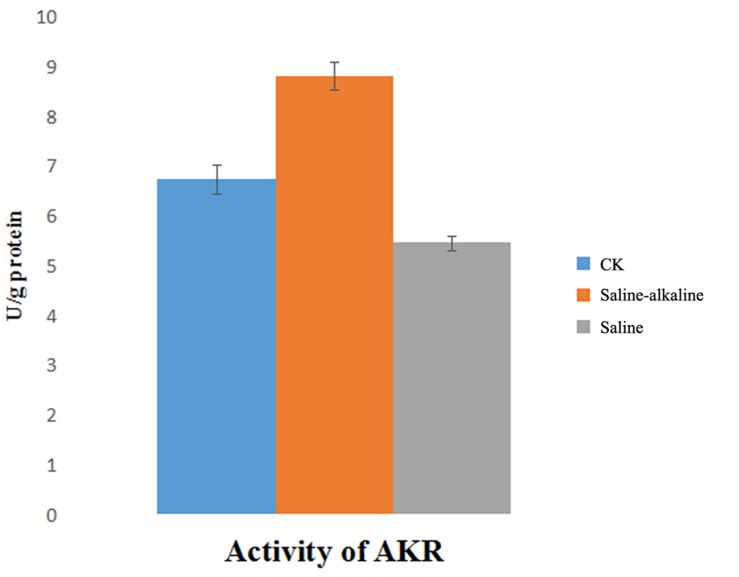
**Activity of AKR in root part under saline stress and saline-alkaline stress respectively**.

Changes in AKR activity were determined after exposure to saline and saline-alkaline stress for 7 days. The activity of AKR was markedly different under both stresses, and it was higher under saline-alkaline stress than under saline stress (**Figure 8**).

## Discussion

Transcriptome analysis showed that the component of the ROS scavenging pathway was dramatically upregulated under saline-alkaline stress conditions. Flavonoids, which are potential non-enzymatic protective substances against reactive oxygen (Hernández et al., 2009), have a strong response to sodic stress. In addition, the downregulation of RuBP carboxylase suggested that the photosynthesis pathway was inhibited under saline-alkaline stress (Li et al., 2010; Bu et al., 2012). These results indicated that the antioxidant capacity is one of the major mechanisms of tolerance to saline-alkaline stress in alfalfa. Furthermore, other potential stress response pathways may exist. Eight different expression genes were screened from transcriptome for their dramatically different or even opposite expression under the saline and saline-alkaline stress conditions. A member of NAC transcription factors and AKR were downregulated under salt stress, but upregulated under saline-alkaline stress.

Reactive oxygen species accumulate in plant cells during biotic and abiotic stress conditions. Saline-alkaline stress with high pH exacerbate the accumulation of ROS (Li et al., 2010; Gong et al., 2014), such as superoxide anions, H_2_O_2_, hydroxyl radicals, and singlet oxygen, leading to oxidative stress that can damage cellular components such as DNA, lipids, proteins, and sugars (Pandhair and Sekhon, 2006). ROS homeostasis in plants is regulated by a complex machinery of enzymatic and non-enzymatic antioxidants through the enzymatic pathway composed of PODs (Sugimoto et al., 2014). The accumulation of MDA was not changed markedly between day 0 and day 1 of treatment, but it dramatically increased after 7 days of treatment, suggesting an oxidative damage to the plasma membrane. The increasing level of ROS and their effect on membrane indicated that plant tissue was exposed to oxidative stress during the entire treatment period.

Flavonoids are a large family of plant secondary metabolites, which contain strong antioxidants (Pourcel et al., 2006) such as ascorbate (vitamin C) and α-tocopherol (vitamin E). It has long been recognized that salt stress induces flavonoid accumulation (Chalker-Scott, 1999; Winkel-Shirley, 2002). Intracellular flavonoids located in the plasma membrane, chloroplast, vacuole, and nuclei are strongly induced by oxidative stress (Hernández et al., 2009). The expression of flavonoid pathway genes is closely related to the content of intracellular ROS. The substantive activity of flavonoid metabolism suggests that flavonoids play an important role in the saline-alkaline stress tolerance mechanism. In addition, the inhibition of the light pathway was observed.

A decrease in the chlorophyll content suggests a decline of the photosynthetic rate, whereas the downregulation of the light-harvesting protein and RuBP carboxylase suggests the inhibition of the plant photosynthetic rate (Gong et al., 2013). A decline in the plant photosynthetic rate may decrease the accumulation of endogenous ROS and reduce the pressure on the POD system caused by excessive intracellular ROS. Moreover, the inhibition of the plant growth rate and developmental processes can improve plant tolerance to saline-alkaline stress.

Peroxidases are widespread in bacteria, fungi, animals, and plants. They are divided into three categories: class I PODs, intracellular PODs that remove intracellular ROS; class II POD, mainly exist in fungi; and class III PODs, extracellular PODs involved in auxin metabolism, cell elongation, lignin metabolism, and resistance to pathogens (Guida et al., 2014). The activity of POD was highly induced at day 1 of treatment and remained constant until day 7 of treatment, revealing the important role of PODs in response to long-term saline-alkaline stress. Class III PODs show higher expression than other PODs; they participate in ROS elimination when seedlings are exposed to long-term saline-alkaline stress. Previous studies characterized the function of class III PODs, and revealed that they are involved in many biological processes (Almagro et al., 2009), including cell elongation, stress defense, seed germination, and especially polymerization of lignin (Shigeto et al., 2015). The differential response of various species of PODs under saline-alkaline conditions suggests that PODs related to intercellular interaction are more important than those respond to ROS under long-term saline-alkaline stress and allow the resuming of plant growth.

In the present study, alfalfa employed different response mechanisms under saline and saline-alkaline stress at the same concentration of sodium ion (0.1 M) and showed a more dramatic change in common upregulated response genes and milder effect in downregulated genes. Genes involved in stress response pathways showed different responses under the two stresses.

The overexpression of xyloglucan endotransglucosylase/hydrolase promotes the cell wall development and induces cell proliferation and differentiation in the seed germination stage (Miedes et al., 2013). The high pH that accompanies saline-alkaline stress may directly damage the cell wall and the membrane; this damage may be reduced by the high expression of xyloglucan endotransglucosylase/hydrolase.

Aldo/keto reductase is a large family involved in plant stress defense. Most AKRs act by reducing aldehydes and ketones to their respective alcohols using NADPH, or rarely NADH, as a cofactor (Penning, 2004). A previous study indicated that AKRs are downregulated under 100-mM saline stress (Postnikova et al., 2013), but upregulated in response to saline-alkaline stress. The higher content of AKR could rapidly eliminate aldehydes and ketones that were generated by cellular metabolic disorders and high pH. These results indicate that detoxification and anti-oxidation play an important role in alfalfa response to saline-alkaline stress, similar to most extreme environmental factors such as heat, cold, and high level of oxidation stress.

Glutathione *S*-transferase presented the opposite trend in the two different treatments; it was downregulated under saline stress by −1.07, but upregulated under saline-alkaline stress by 4.07. Glutathione *S*-transferase plays an important role in the antioxidant pathway; the high expression of glutathione *S*-transferase is indicative of the high levels of active oxygen in the plant root. A similar trend was detected in the NAC transcription factor (similar to Medtr7g085220.1); it was downregulated under saline stress and upregulated under saline-alkaline stress. NAC is a plant specific transcription factor family with diverse roles in development, stress regulation, and response to biotic and abiotic stresses. Previous studies suggested that the NAC transcription factor participates in the regulation of POD synthesis and may be co-expressed with flavonol synthase (Nakashima et al., 2007). The specific response of NAC may play a role in the cellulose synthase and GST pathway regulation. Future studies on NAC identification may uncover a novel mechanism of plant saline-alkaline stress response. Additionally, DEGs with different expression in a variety of stress conditions may reveal the core mechanism and relative pathways of plant saline-alkaline tolerance. Moreover, further studies will help elucidate how to improve the saline-alkaline stress tolerance of sensitive alfalfa cultivars.

## Author Contributions

C-HG and Y-MA designed the study; Y-MA, L-LS, and Y-RL performed the experiment; Y-JS supplied bioinformatics analysis platform; Y-MA and Y-JS performed the data analysis; C-HG provided scientific expertise; Y-MA wrote the manuscript.

## Conflict of Interest Statement

The authors declare that the research was conducted in the absence of any commercial or financial relationships that could be construed as a potential conflict of interest.

## Footnotes

1 wego.genomics.org.cn/cgi-bin/wego/index.pl

2 http://bioinfo.bti.cornell.edu/cgi-bin/itak/index.cgi
