# Supplementary material for: De Novo Transcriptional Analysis of Alfalfa in Response to Saline-Alkaline Stress
Source: Front Plant Sci. 2016 Jul 4;7:931. doi: 10.3389/fpls.2016.00931 (PMC4931813; doi:10.3389/fpls.2016.00931)
Supplement: Supplementary file 7 [file Data_Sheet_7.DOC]

**Supplementary materials legends**

**Data sheet 1: S1-DEGs** The expression level and gene annotation of different express genes in 1day and 7day. 2,286 (1 d treatment) and 2,233 (7 d treatment) DEGs were found in data respectively.

**Data sheet 2: S2-qRT conformation** Detail of candidate genes used in the validation of transcriptome data in samples treated with alkaline-saline solution for 1 d and 7 d.

**Data sheet 3: S3-GO** Genes presented in Histogram of gene ontology (GO) categories in response to 1 d and 7 d of treatment with saline-alkaline stress.

**Data sheet 4: S4-Response to stress** The most significant differentially expressed genes presented in transcriptome data in response to stress in 1 day and 7 day.

**Data sheet 5: S5-TFs** Transcription factors (TFs) differentially expressed in samples after 1 d and 7 d of saline-alkaline stress

**Data sheet 6: S6-Different to salt** The expression level and gene annotation of eight genes showed significantly different expression profile under saline and saline-alkaline stress for 7 d.
